# Mechanical, chemical, structural analysis and comparative release of PDGF-AA from L-PRF, A-PRF and T-PRF - an in vitro study

**DOI:** 10.1186/s40824-020-00193-4

**Published:** 2020-09-11

**Authors:** Shravanthy Ravi, Muthukumar Santhanakrishnan

**Affiliations:** Department of Peridontology, Faculty of Dental Sciences, Sri Ramachandra Institute of Higher Education and Research, Porur, Chennai, 600116 India

**Keywords:** Platelet concentrates, Platelet-rich fibrin, Advanced platelet-rich fibrin

## Abstract

**Background:**

Platelet concentrates have been popularly used in regenerative periodontal therapy as they are autologous in origin and they provide a supernatural concentration of platelets, growth factors and leukocytes. The release profile of various growth factors is considered important during the various phases of wound healing with the most important being the inflammatory phase where the release of the growth factors help in recruitment of cells and in collagen production. With the more recent modifications of PRF namely A-PRF and T-PRF, the mechanical and chemical degradation properties have also improved. The aim of the present study was to correlate the release profile of PDGF-AA from various forms of platelet concentrates (L-PRF, A-PRF, T-PRF) based on their mechanical and chemical properties.

**Methods:**

Blood samples were drawn from 2 male and 3 female systemically healthy patients between 20 and 25 years of age who were about to undergo periodontal regeneration for PRF preparation. The blood sample was immediately centrifuged using a table top centrifuge (Remi R4C) at 1060 rpm (208 x g) for 14 min for A-PRF preparation, 1960 rpm (708 x g) for 12 min for L-PRF preparation and 1960 rpm (708 x g) for 12 min in titanium tubes for T-PRF preparation. Tensile test was performed using universal testing machine. The in vitro degradation test of the prepared PRF membranes were conducted by placing the PRF membrane in 10 ml of pH 7.4 PBS on an orbital shaker set at 50 rpm. SEM evaluation of the PRF membrane was done under both low and high magnification. In order to determine the amount of released growth factor PDGF-AA at 15 min, 60 min, 8 h, 1 day, 3 days, and 10 days, samples were placed into a shaking incubator at 37 °C to allow for growth factor release into the culture media.

**Results:**

On comparing the three PRF membranes, it was found that T-PRF contained the maximum tensile strength (404.61 ± 5.92 MPa) and modulus of elasticity (151.9 ± 6.92 MPa). Statistically significant differences between the three groups were found on comparing the groups for their mechanical properties. In the degradation test, it was found that the maximum amount of degradation was found in L-PRF (85.75%), followed by A-PRF (84.18%) and the least was found in T-PRF (82.27%). T-PRF released the highest amount of PDGF-AA (6060.4 pg/ml) at early time points when compared to A-PRF (5935.3 pg/ml). While T-PRF had rapid release of PDGF-AA, A-PRF had a sustained release of growth factors released at later time points.

**Conclusion:**

Results from the present study indicate that A-PRF is the most favourable form of platelet concentrate in regenerative periodontal therapy as it has a sustained release of growth factors over time.

## Background

Periodontal reconstruction, which is the ideal goal of periodontal treatment, has been constantly evolving over a period of time. Various methods have been proposed to achieve optimal regeneration. Among these methods, guided tissue regeneration (GTR) and guided bone regeneration (GBR) use barrier membranes to separate the periodontal ligament and bone from the epithelium and connective tissue which allow the former to regenerate the defects [1]. Recently, various growth factors have been studied in periodontal regeneration [2] and it is indicated that they might strongly alter the healing process [3]. The inclusion of growth factors for regeneration has evolved into the concept of tissue engineering with the goal to restore tissue function through the delivery of stem cells, bioactive molecules or synthetic tissue constructs engineered in the laboratory [4]. The tissue engineering approach to periodontal regeneration combines three key elements to enhance regeneration- progenitor cells, scaffold or supporting matrix, signaling molecules [5].

In this scenario, platelet concentrates (PC) have been popularly used in regenerative periodontal therapy as they are autologous in origin and they provide a supernatural concentration of platelets, growth factors and leukocytes (90% platelets and 50% leukocytes when compared to the concentration in natural blood). The concept of using platelet concentrates was developed with the aim of utilizing human blood proteins as a source of growth factors capable of supporting angiogenesis and tissue ingrowth based on the notion that blood supply is a prerequisite for tissue regeneration [6]. PRF (Platelet- rich Fibrin), a second-generation platelet concentrate, was introduced by Choukroun et al. in the year 2001 [7] and has been widely used in periodontal regeneration. PRF is a fibrin meshwork that contains platelets, white blood cells, serum, and concentrated growth factors. More recent modifications of the PRF preparation procedure have led to the development of advanced- platelet rich fibrin (A-PRF) which was developed with the low-speed centrifugation concept (LSCC) that uses lower G- forces to obtain higher growth factor release when compared to PRF [8]. A-PRF preparation is characterized by platelets, leukocytes, circulating stem cells, and endothelial cells concentrated in the fibrin clot. By reducing the centrifuge speed, leukocyte infiltration into the red blood cell fraction is minimized. Furthermore, the red blood cell fraction adjacent to the fibrin clot is not completely eliminated. It also results in earlier vascularization, faster soft tissue growth, more cytokines and release of bone morphogenic proteins (BMPs). The LSCC revolutionized the usage of PRF in periodontal regeneration. Less centrifugation time would reduce cell pull-down by centrifugation g-forces, which increases the total number of cells left contained within the top layer of PRF enabling a higher number of leukocytes “trapped” within the fibrin matrix [8]. Another form of platelet concentrate is T-PRF (Titanium prepared platelet-rich fibrin), which is based on the hypothesis that titanium may be more effective in activating platelets than the silica activators used with glass tubes in Choukroun’s L-PRF method and the clot produced in the titanium tubes was clinically identical compared to glass tubes [9]. However, there is limited evidence in the literature of the effectiveness of the various forms of platelet concentrates used for periodontal regeneration which is largely determined by the mechanical and chemical properties and the release profile of the growth factors of the platelet concentrates. In the present study, the release profile of PDGF -AA was analysed amongst all the available growth factors, as PDGF-AA helps in recruitment of progenitor cells to the site of regeneration [10].

With the more recent modifications of PRF namely A-PRF and T-PRF, the mechanical and chemical degradation properties have also improved. While there have been studies comparing PRF to other commercially available membranes, there is limited evidence in literature correlating the chemical and mechanical properties with the release of growth factors amongst the various forms of platelet concentrates (L-PRF, A-PRF, T-PRF).

Hence, the present study was conducted to correlate the release profile of PDGF-AA (Platelet derived growth factor-AA) from various forms of platelet concentrates (L-PRF, A-PRF, T-PRF) based on their mechanical and chemical properties.

## Materials and methods

2 male and 3 female systemically healthy patients between 20 and 25 years of age were included in the present study based on a specific inclusion and exclusion criteria.

Ethics approval was obtained from the institutional ethics committee (CSP/19/SEP/80/304) and all the patients signed a written informed consent to participate in the study. Three samples of blood were collected from the five patients (one each for L-PRF, A-PRF and T-PRF, 15 total samples).

### Inclusion criteria

Systemically healthy patients aged 20–25 years undergoing periodontal regeneration, patients not on any drugs, non-smokers.

### Exclusion criteria

Patients with systemic diseases, smokers, patients consuming alcohol, patients under anti-coagulant therapy, patients under bisphosphonates, pregnant and lactating women and those who have consumed antibiotics within the last 3 months.

## Methodology

### Preparation of PRF clots

Venous blood was collected via venipuncture of the forearm in the antecubital vein into a 10 ml sterile glass vacuum tube. The blood sample was immediately centrifuged using a table top centrifuge (Remi R4C) at 1060 rpm (208 x g) for 14 min for A-PRF preparation, 1960 rpm (708 x g) for 12 min for L-PRF preparation and 1960 rpm (708 x g) for 12 min in titanium tubes for T-PRF preparation [11]. The clot was separated from the three distinct layers that had formed within the tube. The rpm for centrifugation was calculated with the rotor radius of the centrifuge based on the formula [12]

$$ RPM=\sqrt{\frac{GFORCE}{0.00001118\times ROTOR\ RADIUS}} $$

where the rotor radius of the centrifuge used is 16.5 cm.

### Preparation of mold

A specially designed plexiglass mold was fabricated to make the fibrin specimens identical in size, volume, and figure. The thickness of the mold was 2 mm and the width was 2 mm in the narrow middle part and 6 mm in the larger ends. The length of the mold was 31 mm. The total volume of the mold was 104mm^3^.The narrow region of the neck provided the weakest point where the specimen would break [13].

### Preparing the membrane

**Fig. 1:**
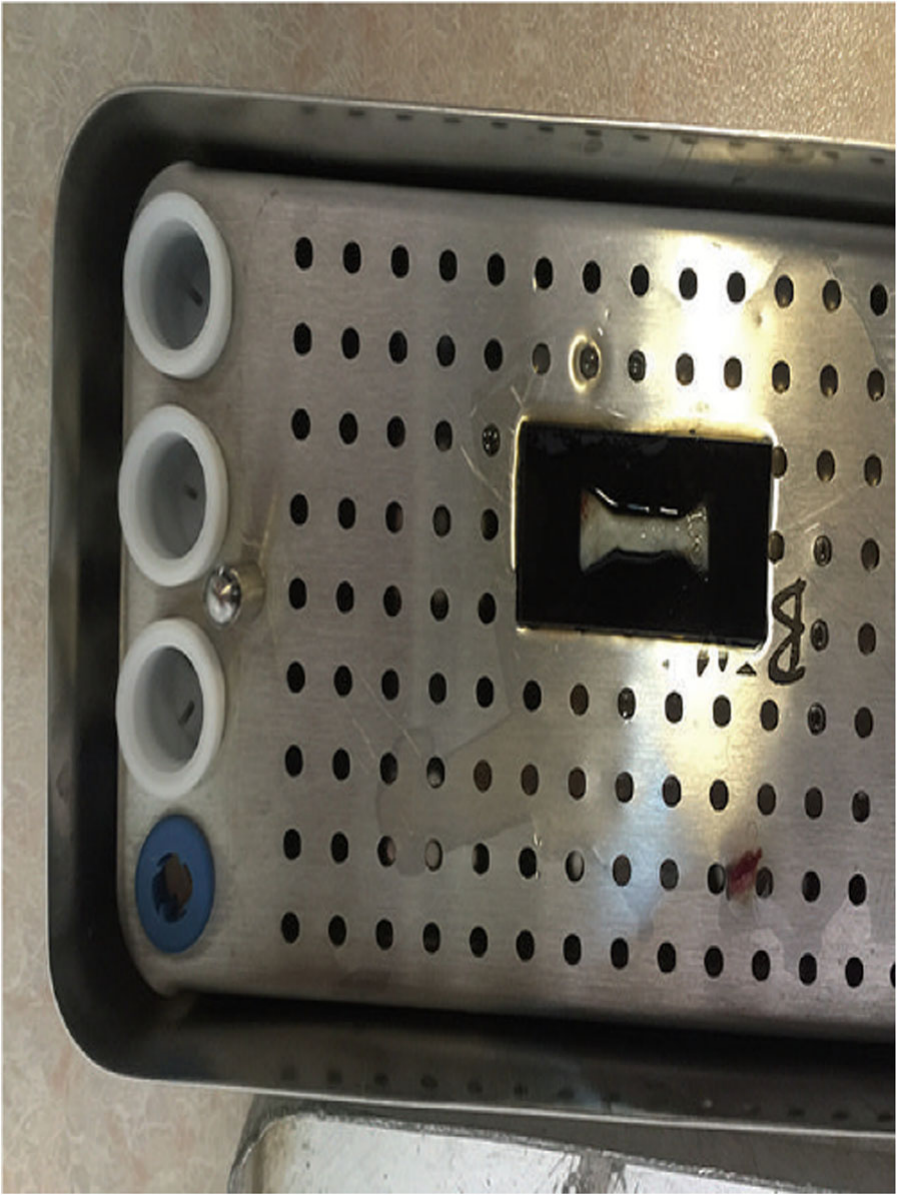
PRF specimen in the mold

The fibrin clot containing platelets in the middle of the tube, between acellular plasma at the top and the red blood cell layer at the bottom was removed from the tube and the attached red blood cells were scraped off and discarded. The early PRF clots were then placed in the mold which was placed on the grid in the PRF Box and covered with the compressor and lid. After 10 min the formed early PRF membrane was prepared and mechanical testing was done to determine the tensile strength and the modulus of elasticity. (Figure 1: PRF specimen in the mold).

### Tensile test

**Fig. 2:**
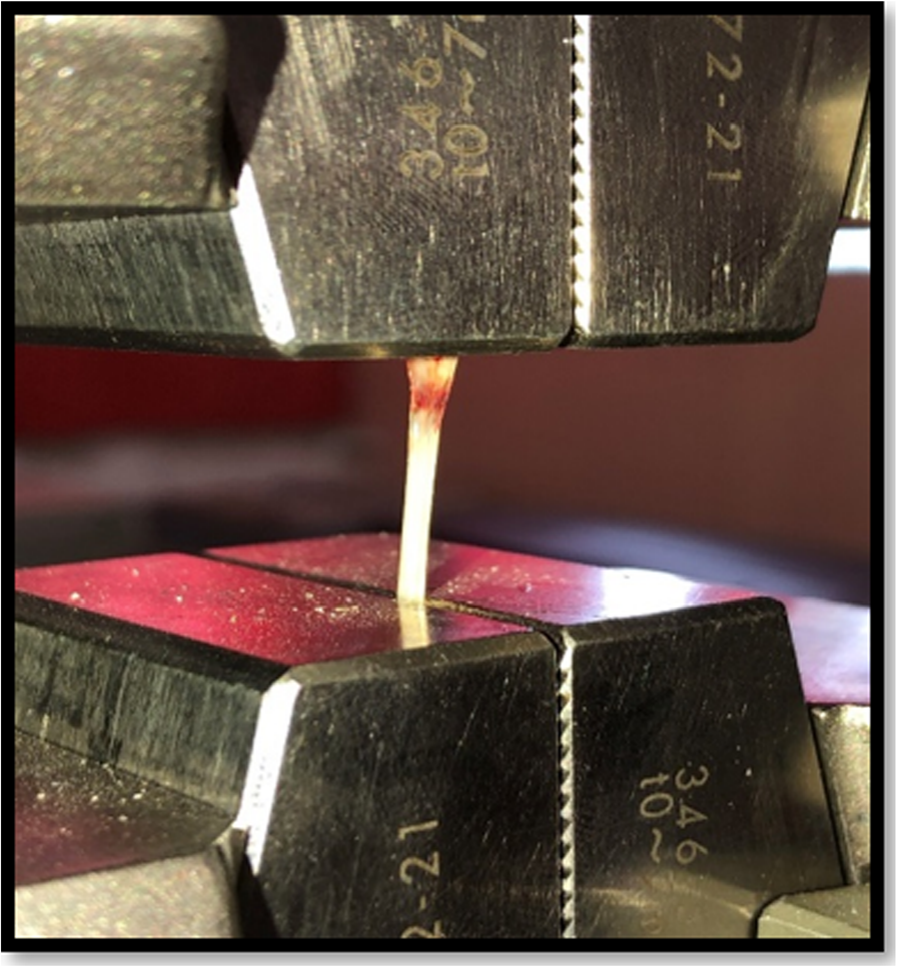
Specimen on the universal testing machine

Tensile test was per formed using universal testing machine. The larger ends of the dog-bone shape specimen were held with the clips of the machine without any tension. Tensile loading was applied at a cross head speed of 1 mm/min; the maximum load at specimen failure was recorded and tensile strength was calculated using the following formula: *S = F / A*, where *F* is maximum force (N) and *A* is unit area (m^2^). Stress-strain curve was recorded with the inbuilt software simultaneously. Stiffness of the specimen (modulus of elasticity) was obtained by stress/strain and the total area under the curve designated as toughness of the specimens [14]. (Figure 2: specimen on the universal testing machine).

### In vitro degradation

The in vitro degradation test of the prepared PRF membranes were conducted by placing the PRF membrane in 10 ml of pH 7.4 PBS on an orbital shaker set at 50 rpm. All the three membranes were weighed on an electronic micro weighing scale at the start of the experiment and at the end of 1-week, the membranes were taken out of the incubation medium, washed with distilled water, dried, and its weight was measured. The degradation profiles were expressed as the accumulated weight losses of the membrane [15].

$$ \% degradation=\frac{initial\ weight- final\ weight}{initial\ weight}\times 100 $$

### Scanning electron microscopic examination

The PRF clots that were compressed in a stainless -steel compressor, were fixed with 2.5% neutralized glutaraldehyde, dehydrated with a series of ethanol solutions and n-butanol, freeze-dried, and then were examined under a scanning electron microscope with an accelerating voltage of 20 kV [16]. Two of the five samples were examined for EDX analysis. Energy Dispersive X-Ray Spectroscopy (EDS/EDX) is a chemical microanalysis technique used in conjunction with SEM. It detects x-rays emitted from the sample during bombardment by an electron beam to characterize the elemental composition of the analyzed volume.

### Growth factor release profile

In order to determine the amount of released growth factor PDGF-AA at 15 min, 60 min, 8 h, 1 day, 3 days, and 10 days, samples were placed into a shaking incubator at 37 °C to allow for growth factor release into the culture media. At each time point, 1 mL of culture media was collected and frozen at − 20 °C. At desired time points, PDGF-AA (DY221, range = 15.63–1000 pg/mL) was quantified using an ELISA (Elabscience) assay. Briefly, 100 μL of assay diluents and 100 μL of sample were incubated for 1.5 h at 37 °C in antibody precoated 96-well plates. The liquid was removed and 100 μL of Biotinylated antibody was added to the wells and was incubated at 37 °C for 1 h. Wells were washed three times with washing buffer and 100 μL of HRP conjugate was added to incubate for 30 min at 37 °C. Wells were then aspirated and washed five times following which 90 μL of Substrate reagent was added for 15 min. 50 μL of Stop solution was added and the absorbance was measured at 450 nm immediately [17].

### Statistical analysis

All data were given as mean ± standard deviation. One-way ANOVA with Bonferroni Post-Hoc test with multiple comparison was done to compare the mean difference among the properties of the membranes. Data was analysed using SPSS software version 21.

## Results

### Macroscopic clot analysis

With respect to size, the clots produced by L-PRF, A-PRF and T-PRF were similar in height and width. However, T-PRF clots weighed the heaviest with a mean weight of 1.81 g while L-PRF weighed 0.9 g and A-PRF weighed 0.9 g.

### Mechanical properties

**Fig. 3:**
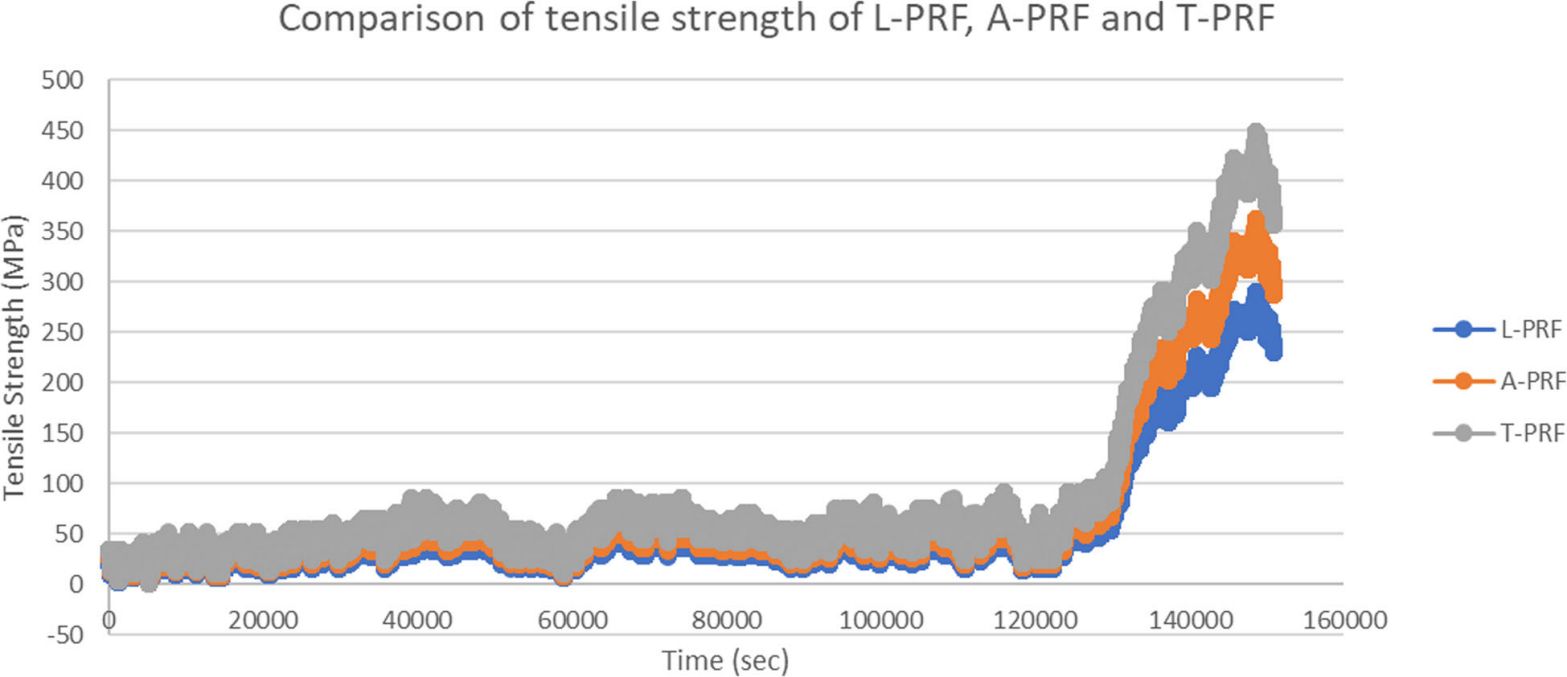
Comparison of tensile strength of the three PRF membranes

A total of five samples were taken for the measurement of the mechanical properties of the PRF membrane and the mean value was computed. On comparing the three PRF membranes, it was found that T-PRF contained the maximum tensile strength (404.61 ± 5.92 MPa) and modulus of elasticity (151.9 ± 6.92 MPa) followed by A-PRF (362.565 ± 5.15 MPa, 122.51 ± 7.15 MPa). It was found that L-PRF contained the least tensile strength (290.076 ± 5.68 MPa) and modulus of elasticity (98.01 ± 7.43 MPa). Table 1 shows the tensile strength and the modulus of elasticity of the PRF membranes. There was statistically significant difference in tensile strength between the three groups. Bonferroni correlation with multiple comparison was done to compare the properties of the membranes and the results were found to be statistically significant as shown in Table 2. (Figure 3: Comparison of tensile strength of the three PRF membranes).

**Table 1:** Tensile strength and modulus of elasticity of the PRF membranes

Sample	Tensile strength (MPa)	Modulus of elasticity (MPa)
L-PRF	290.076 ± 5.68	98.01 ± 7.43
A-PRF	362.565 ± 5.15	122.51 ± 7.15
T-PRF	404.61 ± 5.92	151.9 ± 6.92

**Table 2:** Bonferroni correlation comparing the three PRF membranes

(I) GROUP	(J) GROUP	Mean Difference (I-J)	Std. Error	Sig.	95% Confidence Interval	
					Lower Bound	Upper Bound
L-PRF	A-PRF	−72.48^*^	3.69	.000	−82.76	−62.20
	T-PRF	− 114.53^*^	3.69	.000	− 124.81	−104.25
A-PRF	L-PRF	72.48^*^	3.69	.000	62.20	82.76
	T-PRF	− 42.05^*^	3.69	.000	− 52.33	−31.77
T-PRF	L-PRF	114.53^*^	3.69	.000	104.25	124.81
	A-PRF	42.05^*^	3.69	.000	31.77	52.33

*. The mean difference is significant at the 0.01 level

### Chemical degradation

**Fig. 4:**
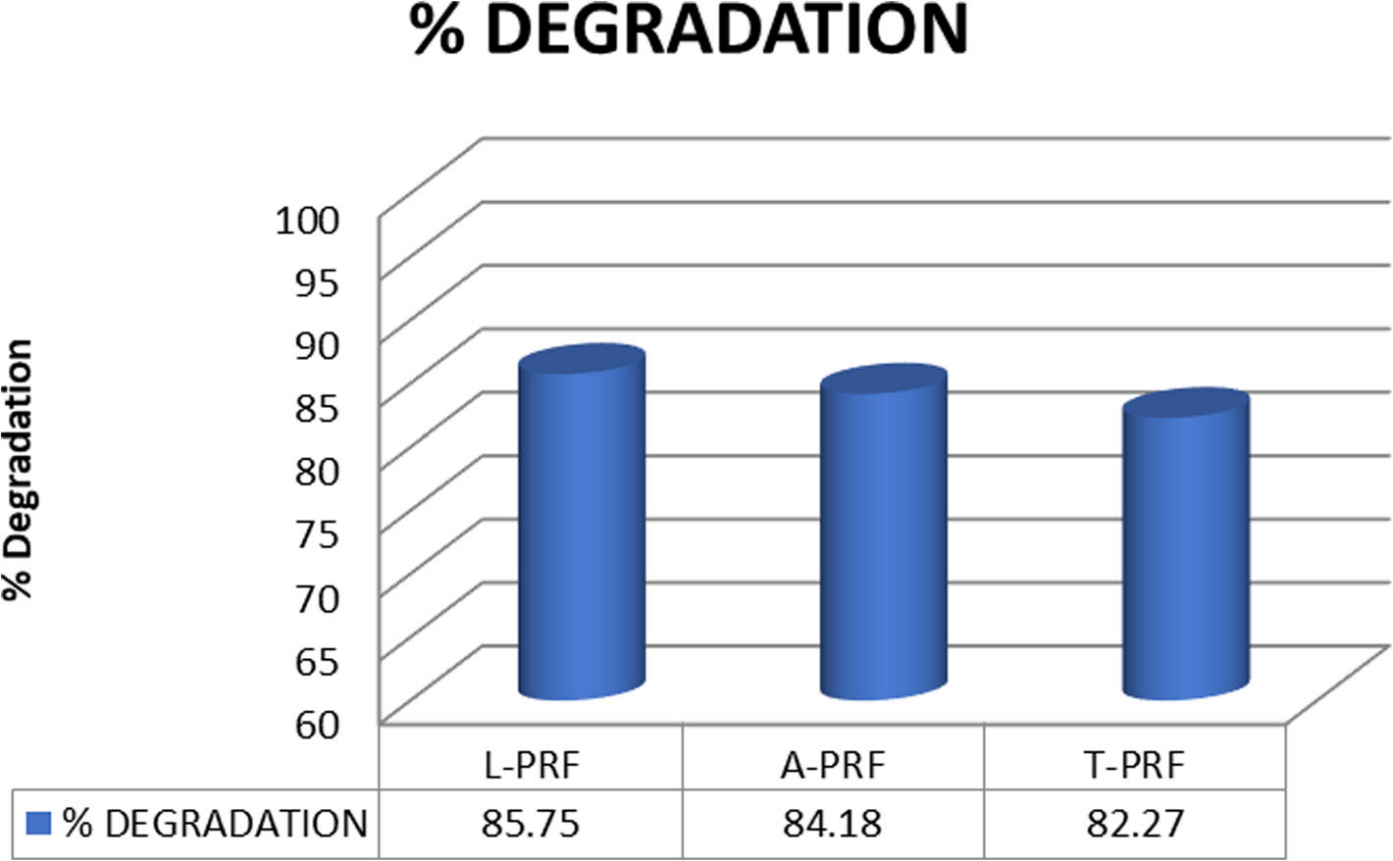
Comparison of degradation percentage of the three PRF membranes show the degradation profiles of the three types of PRF membranes

On placing the three types of PRF membranes in an orbital shaker set, it was noted that all the three PRF membranes degraded to up to 82%. The maximum degradation was found in L-PRF (85.75%), followed by A-PRF (84.18%) and the least was found in T-PRF (82.27%). The difference in percentage of degradation of the three types of PRF was not statistically significant. T-PRF as found to have superior mechanical property and the least degradation rate and L-PRF was found to have the highest degradation rate and the least mechanical property. (Figure 4: Comparison of degradation percentage of the three PRF membranes show the degradation profiles of the three types of PRF membranes). Table 3 shows the degradation data of the PRF membranes.

**Table 3:** Degradation data of the three PRF membranes

	GROUP	MEAN	SD	Mean Rank	*p*-value
PREWEIGHT	L-PRF	0.59	0.56	2.50	0.180
	A-PRF	0.59	.49	2.50	
	T-PRF	1.81	.08	5.50	
	Total	1.00	.71		
POSTWEIGHT	L-PRF	0.07	0.05	2.00	0.116
	A-PRF	0.08	.06	3.00	
	T-PRF	.32	.01	5.50	
	Total	.16	.13		
DEGRADATION	L-PRF	85.75	3.91	5.00	0.156
	A-PRF	84.18	2.02	4.00	
	T-PRF	82.27	.07	1.50	
	Total	84.06	2.51		

The differences were not statistically significant

### Scanning electron microscopic examination

**Fig. 5:**
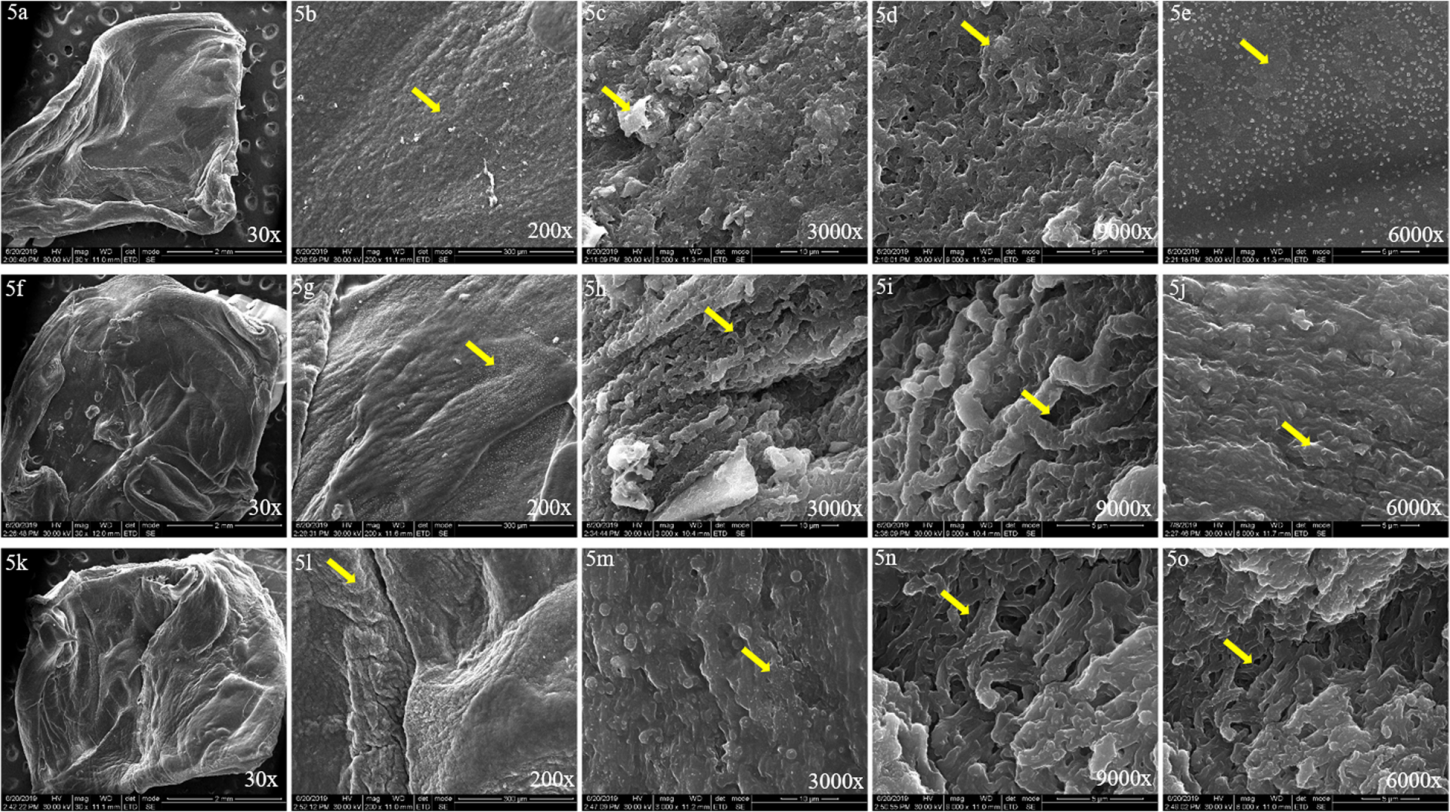
**a-e** L-PRF: 30x, 200x, 3000x, 9000x, 6000x; **f-j** A-PRF: 30x, 200x, 3000x, 9000x, 6000x; **k-o** T-PRF: 30x, 200x, 3000x, 9000x, 6000x

The PRF membranes were freeze-dried and sputter coated with gold prior to scanning electron microscopic examination. (Figure 5a, f, k show the macroscopic structure of the clot following freeze-drying at 30x magnification). Upon SEM examination of L-PRF, it was found that leukocytes and platelets were clumped with a dense matrix that were present close to each other (Fig. 5c: SEM image of L-PRF showing leukocytes and platelets that are clumped (3000x)). These matrix was densely packed resulting in a dense L-PRF membrane with very little space for angiogenesis (Fig. 5d: SEM image of L-PRF showing ill-formed matrix that are densely packed with clumped cells (9000x)). On SEM examination of A-PRF it was found that the leukocytes and platelets were well distributed along the entire surface of the membrane and was not seen as clumps (Fig. 5g: SEM image of A-PRF showing cells that are evenly distributed along the surface of the membrane (200x),5 h: SEM image of A-PRF showing cells that are evenly distributed along the surface of the membrane (3000x)). The matrix was well formed with some porosity present between them which will allow for blood vessel proliferation (Fig. 5i: SEM image of A-PRF showing well-formed matrix that is loosely packed allowing angiogenesis (9000x)). On SEM examination of T-PRF membranes, it was found that the leukocytes and platelets were well distributed along the surface of the membrane (Fig. 5m: SEM image of T-PRF showing well distributed leukocytes and platelets with clumped red cells (3000x)). RBCs were found to be aggregated along the edges of the membrane. The matrix was well-formed and the leukocytes and platelets were well distributed (Fig. 5o: SEM image of T-PRF showing well-formed matrix that is loosely packed allowing angiogenesis (6000x),5n: SEM image of T-PRF showing well-formed matrix that is loosely packed allowing angiogenesis (9000x)). Upon elemental examination of the three types of PRF membranes, it was found that 0.1% of silica was present in L-PRF, 0.5% in A-PRF while T-PRF contained 0.1% of titanium in the place of silica (Table 4). (Figure 5: 5a-e: L-PRF: 30x, 200x, 3000x, 9000x, 6000x; 5f-j: A-PRF: 30x, 200x, 3000x, 9000x, 6000x; 5 k-o: T-PRF: 30x, 200x, 3000x, 9000x, 6000x). Figure 5 shows formed matrix (5d, 5i, 5n) which is the key architecture of PRF along with platelets and leukocytes. (5c, 5 g, 5j, 5 m, 5o).

**Table 4:** Edax data of L-PRF, A-PRF showing the presence of silica and T-PRF showng titanium

	L-PRF		A-PRF		T-PRF	
Element	Wt%	At%	Wt%	At%	Wt%	At%
***CK***	64.94 ± 1.15	71.51 ± 1.2	61.32 ± 2.31	69.58 ± 2.1	61.83 ± 2.35	70.72 ± 1.89
***NK***	11.85 ± 2.1	11.19 ± 1.82	09.20 ± 2.13	08.95 ± 1.92	10.28 ± 1.87	10.08 ± 1.65
***OK***	18.93 ± 1.65	15.65 ± 1.9	19.89 ± 1.21	16.94 ± 1.32	15.72 ± 1.32	13.50 ± 1.27
***NaK***			03.45 ± 0.87	02.04 ± 0.79	04.36 ± 0.98	02.61 ± 0.61
***SiK***	**00.19 ± 0.01**	**00.09 ± 0.01**	**00.59 ± 0.01**	**00.29 ± 0.01**		
***SK***	01.00 ± 0.21	00.41 ± 0.1	01.39 ± 0.1	00.59 ± 0.1	01.60 ± 0.36	00.69 ± 0.13
***ClK***	02.95	01.10	04.17 ± 0.71	01.60 ± 0.76	05.90 ± 0.57	02.28 ± 0.29
***CaK***	00.13	00.04	0.57	0.18		
***TiK***					**00.14 ± 0.01**	**00.04 ± 0.01**

### Growth factor release profile

**Fig. 6:**
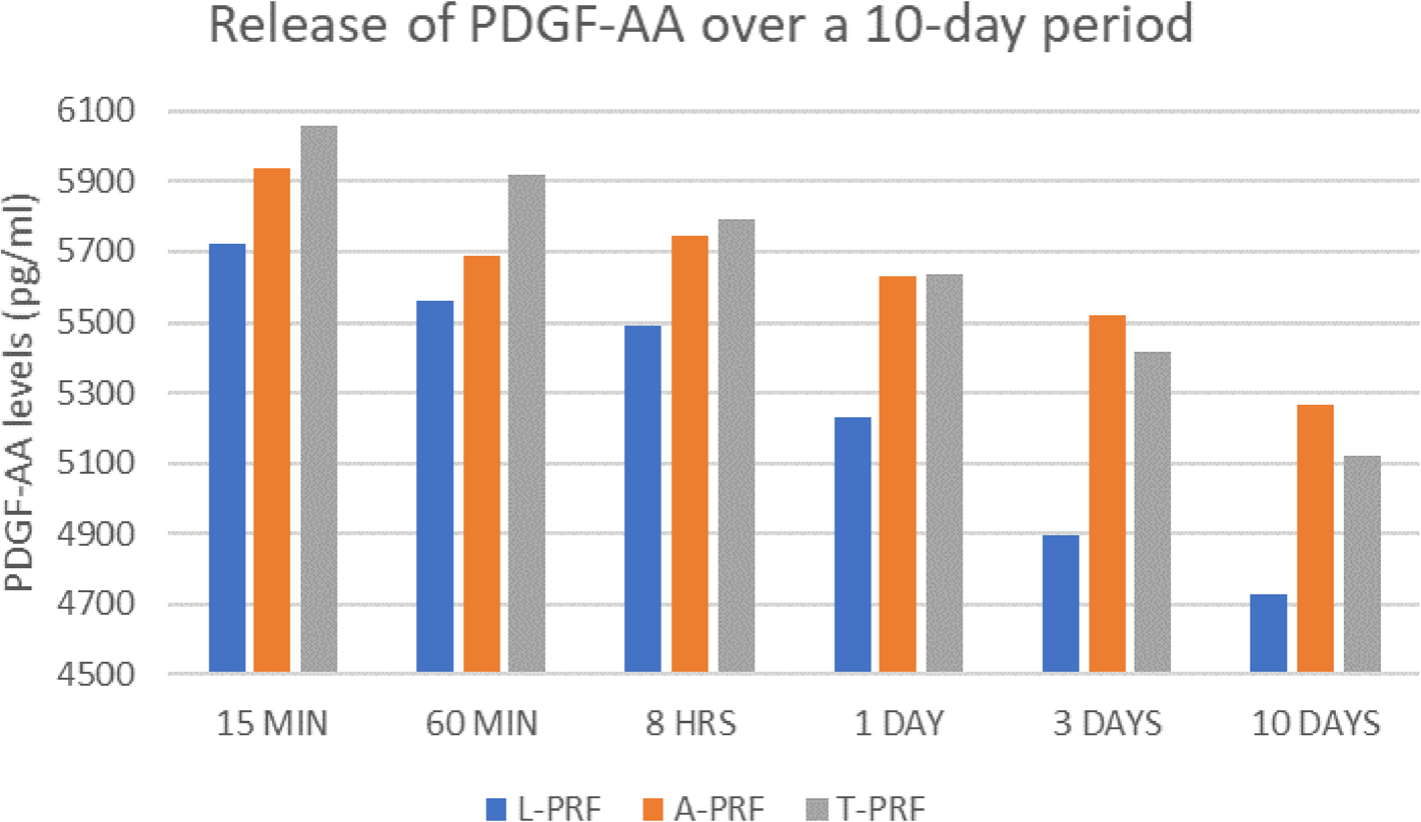
PDGF-AA release profile over a 10-day period

The collected culture media was stored at − 20 °C until further analysis. PDGF-AA growth factor release was analyzed using ELISA. It was found that after 15 min, significantly higher levels of PDGF-AA was released from T-PRF (6060.4 pg/ml) when compared to L-PRF (5721.3 pg/ml) and A-PRF (5935.3 pg/ml). This increased levels of PDGF-AA from T-PRF was observed till day 1, following which the levels of PDGF-AA decreased. A-PRF showed a sustained release of PDGF-AA over the 10-day period (Table 5). For the samples collected on day 1, it was found that there was only a marginal increase of T-PRF (5637.9 pg/ml) when compared to A-PRF (5629 pg/ml). On day 3 and day 10, it was found that the levels of A-PRF were higher than that of L-PRF and T-PRF (Fig. 6: PDGF-AA release profile over a 10-day period). Intergroup comparison of the PRF membranes did not show any statistically significant difference between the various platelet concentrates at different time points. However, the trend showed an increased release of PDGF-AA in T-PRF group at earlier time points and in A-PRF group at later time points. On intragroup comparison, earlier time points showed greater release than at later time points in all groups.

**Table 5:** PDGF-AA release profile over a 10-day period

	L-PRF (pg/ml)	A-PRF (pg/ml)	T-PRF (pg/ml)
15 MIN	5721.33 ± 76.63	5935.36 ± 71.61	6060.44 ± 77.85
60 MIN	5559.12 ± 47.35	5687.67 ± 27.16	5917.77 ± 26.36
8 HRS	5489.02 ± 40.60	5748.52 ± 35.50	5791.25 ± 36.23
1 DAY	5233.24 ± 27.76	5629.08 ± 27.62	5637.99 ± 27.54
3 DAYS	4898.25 ± 19.79	5518.92 ± 22.34	5417.96 ± 23.99
10 DAYS	4730.75 ± 4.34	5266.75 ± 5.45	5124.08 ± 5.29

## Discussion

The recent advancements regarding the use of platelet concentrates have been hypothesized to improve tissue regeneration. However, there have been limited studies evaluating the effectiveness of the PRF membranes in periodontal regeneration in terms of release profile of growth factors [17]. The advent of low-speed centrifugation concept has rendered A-PRF with a three-dimensional architecture which has an increased number of cells including platelets and leukocytes (90% platelets and 50% leukocytes) when compared to their concentration in whole blood which help in release of higher concentration of growth factors including PDGF, VEGF, IGF, FGF, TGF-β when compared to L-PRF. This reduction of G-force also resulted in increased levels of cytokines which help in cellular recruitment and monocytes that help in osteoblastic differentiation. The leukocytes in PRF help in elimination of necrotic debris thereby preventing infection at the defect site. They also help in recruitment of undifferentiated mesenchymal cells native to the site being regenerated and totipotent cells from the cambial layer of the periosteum by upregulation of cytokines and chemokines [18]. Major advances have been recognized over recent years in the architectures of PRF growth factor delivery systems that allow the controlled release of growth factors in a well-ordered manner. In view of these advancements, the applications of PRF are diverse encompassing a number of procedures like sinus floor elevation, ridge augmentation, socket preservation, root coverage, intrabony defects, and furcation defects. It has been shown that fibrin membranes could be better scaffolds for proliferation of periosteal and osseous cells than collagen membranes in vitro [19, 20]. To check the bioavailability of the platelet concentrates during the stages of wound healing, the role of the platelet concentrate should be studied over time corresponding to the proliferation and maturation stages of wound healing which corresponds to 10–14 days. Its use as a membrane is limited to its effect in initial wound healing process. However, the effectiveness of the ideal form of platelet concentrates in regenerative periodontal therapy has not been evaluated. There have been limited studies evaluating the release profile of growth factors based on its mechanical and degradation properties. In this scenario, the present study was done to determine the effectiveness of release profile of PDGF-AA from various forms of PRF (L-PRF, A-PRF, T-PRF) based on its mechanical and chemical properties.

The specimens of this study were selected from healthy individuals with the age range of 20–25 to prevent possible bias from variations in blood components of different sexes, ages, and systemic conditions and platelet index was taken at baseline and compared between the individuals (MPV- 9.9 ± 1.02, PDW- 0.26 ± 0.05) to prevent variations due to the number of platelets at baseline and the release profile [13, 21, 22].

For comparing the mechanical properties of the membranes, the dog-bone-shape mold was used to make the specimens identical in size, volume, and shape as per the protocol suggested by Alston et al. [13] Measuring the tensile strength and the modulus of elasticity was done using the universal testing machine. The universal testing machine was calibrated with the change in dimension of the membrane to be read at every 1 mm/min. The results of the present study demonstrated maximum tensile strength and modulus of elasticity for T-PRF followed by A-PRF. It was found that L-PRF contained the least tensile strength (290.076 ± 5.68 MPa) and modulus of elasticity. Statistically significant differences between the three groups were found on comparing the groups for their mechanical properties. These results could not be compared with other studies as this the first study to our knowledge comparing the mechanical properties of T -PRF. A comparison for the modulus of elasticity of L-PRF with Sam et al. [15] produced a modulus of elasticity of 0.35GPa, the present study showed a lesser modulus of elasticity which was concurrent. In the study done by Khorshidi et al. [14] with L-PRF membrane, the tensile strength was found to be 0.2 MPa. These results were not concurrent with the results of the present study due to difference in protocol where they have measured the tensile strength using a nanoindenter [15].

The structural integrity of the implanted bio absorbable barrier membrane should be preserved for a sufficient time to ensure desired results. Hence to evaluate the degradation time of different platelet concentrates, analysis of chemical degradation was done by placing the membranes in 10 ml of pH 7.4 PBS for a period of 7 days and the maximum amount of degradation was found in L-PRF, followed by A-PRF and the least amount of degradation was found in T-PRF. The difference in percentage of degradation of the three types of PRF was not statistically significant. An association between the mechanical property and chemical degradation rate was found in all the three samples wherein, T-PRF was found to have the highest value for mechanical property and the least degradation, and L-PRF was found to have the highest degradation and the least value for mechanical property. Though the results were not statistically significant, the trend clearly demonstrated the superiority of T-PRF over L-PRF and A-PRF. The results of this study could not be compared to other studies as this is the first study to our knowledge comparing the chemical degradation of T-PRF. A study by Sam et al. [15] showed a degradation percentage of 36% in a solitary sample of L-PRF of its initial weight. These results were not concurrent with the results of the present study. This could be due to non-standardization of the sample as they have not correlated with the platelet index values.

On viewing the specimens under scanning electron microscope, it was found that in L-PRF, leukocytes and platelets were clumped with dense matrix formation. These results were consistent with those obtained by Sam et al. [15] where they found that the junction between the red part and the yellow part of the fibrin clot (buffy coat area) showed spherical structures with an irregular surface which can possibly be identified as leukocytes. There was also a dense aggregate of activated platelets resting on a mature fibrin background in the buffy coat area. Beyond the buffy coat area there were many areas of dense clusters of platelets formed due to extensive aggregation and clotting. Platelet morphology was totally modified by aggregation and clotting processes. These findings were consistent with those reported by Dohan et al. [23] who reported with increasing numbers of platelets and leukocytes along the buffy coat area. In A-PRF, leukocytes and platelets were well distributed along the entire surface of the membrane and was not seen as clumps. The matrix was well formed with some porosity present between them which will allow for blood vessel proliferation. These results were consistent with the findings produced by Isobe et al. [16] where it was stated that A-PRF clots contained mature fibrin threads. In T-PRF, it was found that the leukocytes and platelets were well distributed along the surface of the membrane. RBCs were found to be aggregated along the edges of the membrane. The matrix was well-formed and the leukocytes and platelets were well distributed. These results were consistent with the findings of Tunali et al. [9, 24] where they demonstrated a well-organized matrix and matured fibrin network in the T-PRF clot.

Growth factor release is a key function of these fibrin clots for tissue regeneration. The release profile of growth factors was assessed as it corresponds to the proliferative and maturation phase of wound healing. The present study analysed the release profile of PDGF -AA as amongst all the available growth factors, PDGF-AA helps in recruitment of progenitor cells to the site of regeneration [10]. In order to determine the amount of released growth factor PDGF-AA at 15 min, 60 min, 8 h, 1 day, 3 days, and 10 days, samples were placed into a shaking incubator at 37 °C to allow for growth factor release into the culture media and was quantified using an ELISA assay. The results of the present study showed that after 15 min, significantly higher levels of PDGF-AA was released from T-PRF when compared to L-PRF and A-PRF. This increased levels of PDGF-AA from T-PRF was observed till day 1, following which the levels of PDGF-AA decreased. A-PRF showed a sustained release of PDGF-AA over the 10-day period. Though there was no statistically significant difference between the group at various time points, the trend showed increased PDGF-AA at earlier time points in T-PRF group and in A-PRF group at later time points. This could probably be due to the increase in growth factors which is proportionate to the increase in the number of cells (90% platelets and 50% leukocytes) due to the LSCC concept [8, 25]. The A-PRF clot showed a more porous structure with a larger interfibrous space compared to that of PRF which resulted in more accumulation of cells and its sustained release. The results of the study concurred with the findings of Kobayashi et al. [17] who demonstrated that, PRP had a rapid release of growth factors and A-PRF produced better release over time. The release pattern of T-PRF could not be compared with other studies as this is the first study to our knowledge to investigate the release of growth factors in T-PRF. T-PRF has comparable mechanical and chemical properties and a similar surface morphology to A-PRF which could be attributed to the incorporation of titanium particles. The differential release profile showed increased levels of PDGF-AA in T-PRF at early time points and decreased levels at later time points when compared to A-PRF. This could be attributed to the reduced G-force in A-PRF resulting in an increased cellular content (platelets and WBCs) which could be attributed to the sustained release over time.

The present study is the first study to our knowledge comparing the mechanical properties, chemical properties and growth factor release over time of various types of platelet concentrates with T-PRF. The results of the present study clearly demonstrated the superiority of A-PRF when it comes to the sustained release of growth factors (PDGF-AA) which could prove beneficial in regenerative periodontal therapy. However, the present study had the limitation of evaluating the release profile of only one growth factor (PDGF-AA). Future studies are required to compare the in-vivo evaluation of release profile of all the growth factors present in platelet concentrates and evaluation of behaviour of various cell types following the application of various forms of platelet concentrates which would give a clearer indication of choosing the appropriate form of platelet concentrates based on different clinical scenarios.

## Conclusion

Within the limitations of the present study, it can be stated that A-PRF is the most favourable form of platelet concentrate in regenerative periodontal therapy as it has a sustained release of growth factors over time, which corresponds to the hemostatic and inflammatory phase of wound healing.

## Data Availability

Available.

## Abbreviations

A-PRF: Advanced-platelet rich fibriGGGn
ELISA: Enzyme-linked immunosorbent assay
FGF: Fibroblast growth factor
GBR: Guided bone regeneration
GTR: Guided tissue regeneration
IGF: Insulin-like growth factor
L-PRF: Leucocyte- and Platelet -rich fibrin
LSCC: Low speed centrifugation concept
PBS: Phosphate-buffered saline
PC: Platelet concentrates
PDGF-AA: Platelet derived growth factor -AA
PRF: Platelet - rich fibrin
RBC: Red blood cells
RPM: Revolutions per minute
SEM: Scanning electron microscopy
TGF-β: Transforming growth factor - beta
T-PRF: Titanium-prepared platelet rich fibrin
VEGF: Vascular endothelial growth factor
